# DIA-Based Quantitative Proteomics Reveals Adaptive Responses and Potential Mechanisms of Se(IV) Resistance in *Rhodococcus qingshengii* PM1

**DOI:** 10.3390/microorganisms14071455

**Published:** 2026-07-01

**Authors:** Zhikang Guo, Zecheng Li, Fang Chen, Mu Peng, Haibo Wang

**Affiliations:** 1 Hubei Key Laboratory of Biological Resources Protection and Utilization, Hubei Minzu University, Enshi 445000, China; 15072395917@163.com (Z.G.); 15545428880@163.com (Z.L.); 2024322040039@stu.scu.edu.cn (F.C.); pengmu1025@hotmail.com (M.P.); 2 College of Biological and Food Engineering, Hubei Minzu University, Enshi 445000, China; 3 College of Life Science, Sichuan University, Chengdu 610065, China

**Keywords:** selenite reduction, *Rhodococcus qingshengii*, DIA proteomics, ferredoxin, redox homeostasis, oxidative stress

## Abstract

Microbial reduction of soluble selenium oxyanions is a sustainable strategy for remediating selenium-contaminated environments, yet the molecular mechanisms underlying selenite tolerance in the genus *Rhodococcus* remain poorly understood. In this study, we investigated the proteomic adaptation of the highly tolerant strain *Rhodococcus qingshengii* PM1 under high-concentration selenite stress (50 mM Na_2_SeO_3_) using a data-independent acquisition (DIA)-based quantitative proteomics approach. A total of 3335 proteins were identified, and 3310 proteins were retained for downstream analysis. Comparative proteomics revealed 1411 differentially expressed proteins, including 972 upregulated and 439 downregulated proteins in the selenite-treated group. These changes indicate extensive systems-level proteomic reprogramming and support a growth–defense trade-off strategy. Strain PM1 strongly upregulated ferredoxin and multiple respiratory-chain- and oxidoreductase-associated proteins, suggesting a ferredoxin-associated electron-transfer network that may contribute to Se(IV) transformation and intracellular redox adjustment. In parallel, proteins involved in sulfur assimilation, cysteine/methionine and selenocompound metabolism, ergothioneine biosynthesis, GSH-associated metabolism, Trx/MSH thiol-redox systems, peroxidase/Ohr-Prx detoxification, metalloid/oxyanion resistance, urease-associated pH adaptation, DNA repair, and cell-envelope remodeling were induced, indicating activation of multilayered defense and homeostasis mechanisms. Conversely, proteins associated with central carbon metabolism, carbohydrate uptake, and ribosome-dependent translation were repressed, suggesting reduced growth investment and energy conservation under severe selenite pressure. Overall, this study provides a systems-level proteomic framework for understanding Se(IV) resistance in *R. qingshengii* PM1 and identifies candidate targets for future functional validation, strain engineering, and selenium/metal(loid) bioremediation.

## 1. Introduction

As an essential trace element, selenium (Se) is incorporated into biological systems in multiple chemical forms, such as selenocysteine, and plays a pivotal role in maintaining redox homeostasis and supporting normal physiological development [1]. However, Se exhibits an exceptionally narrow physiological safety margin, rendering the boundary between its beneficial and toxic effects indistinct [2]. Although recommended intake levels vary among institutions, excessive Se consumption has been demonstrated to induce severe physiological toxicity [3]. Beyond nutritional exposure, natural geochemical processes such as rock weathering, together with anthropogenic activities including mining and metal smelting, have led to the persistent accumulation of inorganic Se in aquatic and soil environments [4]. Such accumulation increases ecological and human health risks through bioaccumulation and trophic transfer along aquatic food chains [5], and elevated selenium can impose both acute and chronic toxicity on aquatic organisms [6].

In response to Se contamination, remediation strategies have increasingly shifted from conventional physicochemical approaches, such as adsorption and chemical precipitation, toward more sustainable bioremediation technologies [7]. Microbial Se reduction is of particular interest because it can transform soluble and highly toxic Se oxyanions, including Se(IV) and Se(VI), into less soluble and generally less toxic elemental Se nanoparticles (SeNPs) [8]. This transformation not only contributes to contaminant detoxification but also offers potential for resource recovery, as biogenic SeNPs exhibit distinctive physicochemical and biological properties [9]. To date, Se reduction mechanisms have been investigated in several model or representative bacterial genera, including *Pseudomonas* [10] and *Bacillus* [11]. Nevertheless, microorganisms with stronger stress tolerance and broader metabolic versatility are still needed for the treatment of high-Se environments.

The genus *Rhodococcus* comprises widely distributed, high-G+C actinomycetes known for their exceptional catabolic capacity and environmental adaptability [12]. Rhodococcus species have shown strong potential in the biodegradation of diverse organic pollutants, including hydrocarbons [13], polychlorinated biphenyls (PCBs) [13] and polyurethane monomers [14]. However, compared with their well-documented role in organic pollutant degradation, the metabolic behavior of *Rhodococcus* under inorganic selenium stress remains poorly understood [15]. Although adaptive responses to selenite have been reported in other actinomycetes such as *Kitasatospora* [16], the molecular network by which *Rhodococcus* tolerates and transforms selenite has not been systematically elucidated. In our previous work, a highly selenite-tolerant strain, *R. qingshengii* PM1, was isolated from a selenium-rich mining area, and its genomic features were preliminarily characterized [17,18]. However, genomic information mainly reflects metabolic potential, whereas stress adaptation and detoxification depend on the functional execution of proteins under defined environmental conditions.

Proteomics provides a direct approach for capturing cellular functional responses to environmental stress because changes in protein abundance more closely reflect cellular phenotype and metabolic activity than genomic potential alone [19]. In particular, data-independent acquisition (DIA)-based quantitative proteomics has emerged as a high-throughput, accurate, and reproducible strategy for large-scale proteome quantification and has been widely applied to investigate stress-response mechanisms [20,21]. DIA-based proteomic approaches have revealed molecular response signatures in bacteria under diverse physiological or stress-related conditions, including adhesion/invasion-associated responses in *Cronobacter sakazakii* [22], cold- and acid-stress responses in *Lactiplantibacillus plantarum* [23], and external-stress responses in *Bacillus cereus* spores [24]. Despite the known metabolic versatility of *Rhodococcus*, the proteomic remodeling that enables survival under high-concentration selenite stress remains unclear.

Here, we used DIA-based quantitative proteomics to characterize proteome remodeling in *R. qingshengii* PM1 under severe selenite stress and to identify candidate pathways associated with selenite tolerance and reduction. By analyzing differentially expressed proteins and enriched metabolic pathways, this study aims to reveal the metabolic reprogramming strategies that support strain PM1 survival and detoxification under selenium stress, thereby providing a theoretical basis for the development of *Rhodococcus*-based bioremediation technologies for Se-contaminated environments.

## 2. Materials and Methods

### 2.1. Microbial Strain and Culture Conditions

The selenite-reducing strain *R. qingshengii* PM1 was isolated from selenium-rich carbonaceous mudstone soil in Yutangba, Enshi, Hubei, China [17], and its complete genome sequence has been deposited in GenBank under accession number CP104782, which is associated with BioProject accession number PRJNA881770. Its taxonomic identity and selenite-reducing capacity were previously confirmed through physiological, biochemical, and phylogenetic analyses [18].

For proteomic analysis, the inoculum was prepared by cultivating strain PM1 in LB broth at 28 °C with shaking at 160 rpm until the OD_600_ reached approximately 1.0. The resulting culture was used as the seed culture and inoculated into fresh LB broth at 1% (*v*/*v*), resulting in an initial OD_600_ of approximately 0.01. The experimental cultures were then grown either without selenite or with 50 mM Na_2_SeO_3_ at 28 °C with shaking at 160 rpm. The concentration of 50 mM Na_2_SeO_3_ was selected based on previous physiological and biochemical characterization of strain PM1, which showed that this strain could tolerate up to 100 mM Na_2_SeO_3_ and reduce 50 mM sodium selenite by 99% within 72 h [18]. Moreover, the same Na_2_SeO_3_ concentration has been used in previous transcriptomic and metabolomic analyses of strain PM1, enabling direct comparison and integrative interpretation of the present proteomic data with previously reported multi-omics results [25]. Cells were harvested at approximately 15 h when the OD_600_ reached approximately 1.0. Three independent biological replicates were prepared for each group and subjected to DIA-based proteomic analysis.

### 2.2. Protein Extraction and Digestion

Proteomic analysis was performed by Majorbio Bio-Pharm Technology Co. Ltd. (Shanghai, China). Frozen bacterial samples were suspended in protein lysis buffer containing 8 M urea, 1% SDS, and a protease inhibitor cocktail (Bimake, #B14002). The use of urea and SDS was intended to improve protein denaturation and solubilization; however, no specific membrane-protein enrichment or membrane-fractionation procedure was performed. Therefore, highly hydrophobic or tightly membrane-associated proteins may have been less efficiently recovered than soluble cytoplasmic proteins. The samples were disrupted using a Wonbio-384CL tissue grinding system (Shanghai Wonbio Biotechnology Co., Ltd., Shanghai, China) three times for 180 s each, followed by non-contact low-temperature ultrasonic treatment using an SBL-22DT ultrasonic thermostatic cleaner (Ningbo Scientz Biotechnology Co., Ltd., Ningbo, China) for 30 min. After centrifugation at 14,000× *g* for 15 min at 8 °C, the supernatant was collected, and protein concentration was determined using the bicinchoninic acid (BCA) assay according to the manufacturer’s instructions. Protein quality was further assessed by SDS-PAGE.

For each sample, 100 μg of protein was adjusted to a final concentration of 100 mM triethylammonium bicarbonate buffer (TEAB). Proteins were reduced with 10 mM tris(2-carboxyethyl)phosphine (TCEP) at 37 °C for 60 min and alkylated with 40 mM iodoacetamide at room temperature for 40 min in the dark. Proteins were precipitated with pre-cooled acetone at −20 °C for 4 h, followed by centrifugation at 10,000× *g* for 20 min at 4 °C. The resulting pellet was resuspended in 100 μL of 100 mM TEAB. Trypsin was then added at an enzyme-to-protein ratio of 1:50 (*w*/*w*), and digestion was performed overnight at 37 °C.

### 2.3. Peptide Desalting and Quantification

After trypsin digestion, peptides were dried under vacuum and reconstituted in 0.1% trifluoroacetic acid. The peptides were desalted using HLB cartridges and dried using a vacuum concentrator. Peptide concentration was determined using a NanoDrop One spectrophotometer (Thermo Scientific, Waltham, MA, USA) with the predefined peptide/protein A205 program according to the manufacturer’s instructions. Briefly, 1–2 μL of each peptide solution was loaded onto the pedestal, and peptide concentration was calculated based on absorbance at 205 nm. The peptide concentrations were measured using the Peptide Quantification Kit (Thermo Fisher Scientific, Rockford, IL, USA, cat. no. 23275).

### 2.4. DIA Mass Spectrometry Analysis

Based on peptide quantification, equal amounts of peptides from each sample were subjected to data-independent acquisition mass spectrometry. Peptide separation was performed using a VanquishNeo liquid chromatography system coupled to an Orbitrap Astral mass spectrometer (Thermo Fisher Scientific, Bremen, Germany). Peptides were separated on a uPAC High Throughput column (75 μm × 5.5 cm, Thermo Scientific, Ghent, Belgium). Mobile phase A consisted of water containing 2% acetonitrile and 0.1% formic acid, and mobile phase B consisted of water containing 80% acetonitrile and 0.1% formic acid. Peptides were eluted using an 8 min segmented gradient as follows: 4% B at 0 min, 8% B at 0.1 min, 12.5% B at 1.0 min, 12.6% B at 1.1 min, 22.5% B at 3.6 min, 45% B at 5.8 min, and 99% B from 6.4 to 8.0 min. DIA data were acquired using Xcalibur 4.7 software. The Orbitrap Astral mass spectrometer was operated in positive-ion DIA mode with an ion source voltage of 1.5 kV. The mass scan range was set to 100–1700 *m*/*z*. Full MS1 scans were acquired in the Orbitrap analyzer at a resolution of 240,000, with an absolute AGC target of 5.0 × 10^6^ and a maximum injection time of 3 ms. DIA MS2 scans were acquired in DIA mode using an isolation window of 2 *m*/*z*. MS2 scans were acquired with an absolute AGC target of 5.0 × 10^4^ and a maximum injection time of 3 ms.

### 2.5. Protein Identification and Bioinformatic Analysis

DIA raw data were analyzed using Spectronaut^TM^ software version 19. The protein database used for searching was derived from the *R. qingshengii* PM1 genome sequence (CP104782). The main search parameters were as follows: peptide length, 7–52 amino acids; digestion enzyme, trypsin/P; maximum missed cleavages, 2; fixed modification, carbamidomethylation of cysteine; variable modifications, oxidation of methionine and protein N-terminal acetylation. Protein and peptide identifications were filtered with a false discovery rate of ≤1%. Only proteins that passed the protein-level FDR threshold and had valid quantitative information for comparative analysis were retained for downstream statistical analysis. Proteins lacking reliable quantitative values after quality-control filtering were excluded from subsequent differential-expression analysis. Peptide confidence was set to ≥99%, and the XIC width was set to ≤75 ppm. Protein quantification was performed using the MaxLFQ method. Identified proteins were functionally annotated against the Gene Ontology (GO), Kyoto Encyclopedia of Genes and Genomes (KEGG), EggNOG, Pfam, and COG databases. Subcellular localization was predicted using PSORTb. Protein–protein interaction analysis was performed using STRING v11.5 with a medium confidence threshold of 0.4.

### 2.6. Statistical Analysis

Bioinformatic analysis of proteomic data was performed using the Majorbio Cloud platform (Majorbio Bio-Pharm Technology Co., Ltd., Shanghai, China). Protein abundance values were log2-transformed and normalized prior to statistical analysis. For differential-expression analysis, only proteins with valid quantitative values in both PM1 and PM1Se groups were included in fold-change-based statistical testing. Proteins detected exclusively in one group were not included in fold-change-based DEP identification because reliable abundance ratios could not be calculated in the absence of quantitative values in the other group; instead, these proteins were analyzed separately as group-specific detected proteins. Differentially expressed proteins (DEPs) were identified using a two-tailed Student’s *t*-test. To control the false-positive rate associated with multiple testing, *p* values were adjusted using the Benjamini–Hochberg method, and proteins with |log_2_FC| ≥ 1 and adjusted *p* < 0.05 were defined as DEPs.

GO and KEGG enrichment analyses were performed using the Majorbio Cloud Platform. Enrichment significance was evaluated using a hypergeometric test, and *p* values were adjusted using the Benjamini–Hochberg method. Multivariate analyses, including principal component analysis and correlation heatmaps, as well as data visualization, were performed in R v4.3.1. Data are presented as the mean ± SD of three independent biological replicates.

## 3. Results

### 3.1. Global Overview of DIA-Based Proteomic Profiling Under Selenite Stress

To obtain a global view of the proteomic response of *R. qingshengii* PM1 to selenite stress, we performed a DIA-based comparative proteomic analysis between the control group (PM1) and the selenite-treated group (PM1Se). In total, 3335 proteins were identified, of which 3310 proteins passed the identification and quantification quality-control criteria and were retained for downstream comparative analysis, corresponding to 25,595 peptides (Table S1). Quality-control analysis showed that most identified peptides were distributed between 7 and 20 amino acids and that many proteins were supported by multiple peptides (Figure S1), indicating reliable tryptic digestion and protein identification.

**Figure 1:**
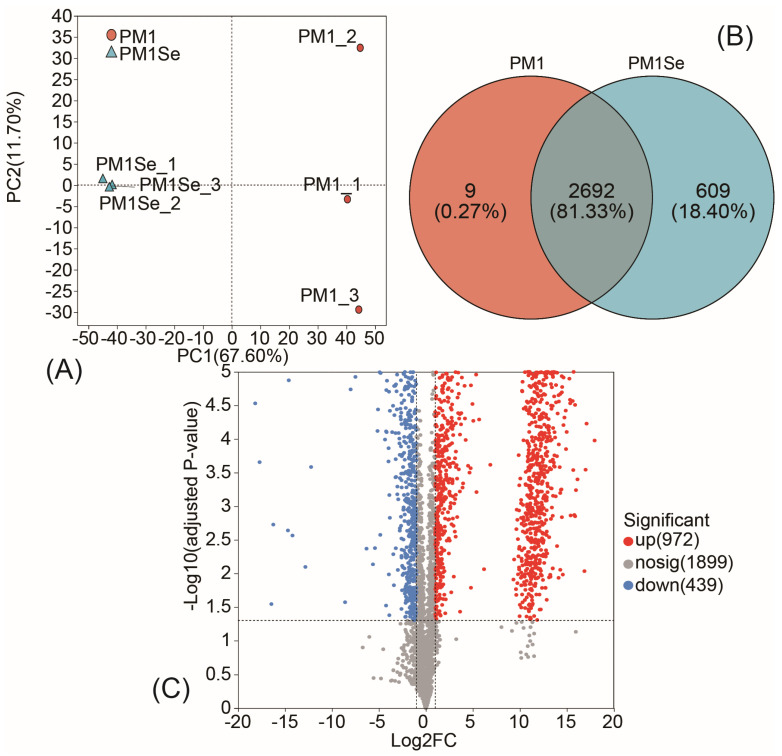
Global proteomic overview of *R. qingshengii* PM1 under selenite stress. (**A**) Principal component analysis (PCA) showing clear separation between the control group (PM1) and the selenite-treated group (PM1Se). (**B**) Venn diagram summarizing the overlap of proteins retained for downstream analysis between the two groups. (**C**) Volcano plot of quantified proteins in PM1Se versus PM1. The x-axis shows log2 fold change (log2FC), and the y-axis shows −log10(Benjamini–Hochberg adjusted *p*-value). Differentially expressed proteins were defined as |log_2_FC| ≥ 1 and Benjamini–Hochberg adjusted *p* < 0.05. Red dots indicate significantly upregulated proteins, blue dots indicate significantly downregulated proteins, and gray dots indicate non-significant proteins.

Principal component analysis (PCA) revealed clear separation between the two groups, with PC1 and PC2 explaining 67.60% and 11.70% of the total variance, respectively, indicating that selenite treatment was the major factor driving proteomic divergence between PM1 and PM1Se (Figure 1A). In addition, the Venn diagram showed a clear difference in protein detection profiles between the two groups (Figure 1B). Among the 3310 proteins retained for downstream analysis, 2692 were shared by both groups, whereas 609 proteins were detected only in PM1Se and 9 were specific to the control group. This pattern suggests that selenite exposure was associated with a broader detectable protein repertoire in PM1Se. Taken together, these results demonstrate the reliability of the DIA-based proteomic dataset and reveal substantial proteomic divergence in PM1 under selenite stress, providing a basis for subsequent differential-expression and functional analyses.

### 3.2. Identification and Overall Distribution of Differentially Expressed Proteins

To identify proteins associated with the response of PM1 to selenite stress, quantified proteins were screened using the criteria of |log2FC| ≥ 1 and Benjamini–Hochberg adjusted *p* < 0.05. Based on this revised threshold, 1411 differentially expressed proteins (DEPs) were identified, including 972 upregulated and 439 downregulated proteins in PM1Se relative to PM1 (Table S2). Compared with the raw *p*-value-based screening result, 69 proteins did not remain significant after Benjamini–Hochberg correction and were therefore excluded from the final DEP list. These results indicate that selenite exposure induced extensive changes in the proteomic profile of PM1. The volcano plot showed an asymmetric distribution of DEPs, with upregulated proteins substantially outnumbering downregulated proteins (Figure 1C). This pattern suggests that selenite stress induced a predominantly upregulated proteomic response, rather than causing a general suppression of protein abundance. The large number of upregulated proteins further indicates that PM1 actively remodels its proteome under severe selenite stress.

### 3.3. Functional Annotation and Pathway Enrichment Analyses of DEPs

**Figure 2:**
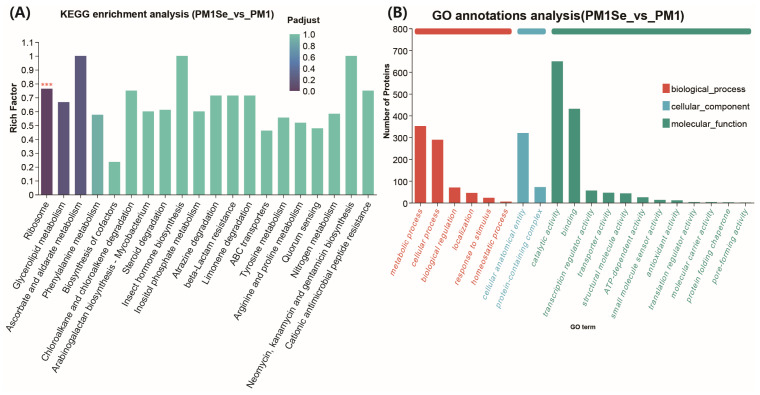
Functional annotation and KEGG pathway enrichment analysis of differentially expressed proteins (DEPs) in *R. qingshengii* PM1 under selenite stress. (**A**) KEGG pathway enrichment analysis of DEPs. The y-axis represents the enrichment factor, and the bar color indicates the adjusted *p* value. (**B**) GO functional classification of DEPs at level 2 across biological process, cellular component, and molecular function categories. Asterisks indicate statistical significance based on adjusted *p* values: ***, adjusted *p* < 0.001.

To characterize the functional response of *R. qingshengii* PM1 to selenite stress, DEPs were subjected to KEGG pathway enrichment and GO functional classification analyses. KEGG enrichment analysis showed that the ribosome pathway was significantly enriched after multiple-testing correction, with the lowest adjusted *p* value among the detected pathways (Figure 2A; Table S3). This result is consistent with the marked changes in translation-related proteins and suggests that protein synthesis was strongly affected under selenite stress.

In addition to the ribosome pathway, glycerolipid metabolism and ascorbate and aldarate metabolism were among the top pathways ranked by raw *p* value and rich factor, although they did not remain significant after Benjamini–Hochberg correction. These pathways may indicate potential involvement of membrane-associated metabolism and oxidative-stress-related metabolism in the response to selenite stress. ABC transporter-related pathways were also detected among the enriched terms, suggesting that transport processes may participate in stress adaptation, but this trend should be interpreted cautiously because of the limited adjusted significance.

GO functional classification showed that DEPs were broadly distributed across the three major categories of biological process, cellular component, and molecular function (Figure 2B; Table S4). Major GO terms included “metabolic process”, “cellular process”, “biological regulation”, “response to stimulus”, “catalytic activity”, “binding”, and “transporter activity”. Cellular component categories were mainly represented by “cellular anatomical entity” and “protein-containing complex”. These results indicate that selenite stress affected a broad range of cellular functions in PM1, particularly metabolism, catalytic activity, binding functions, and translation-associated processes. A GO chord diagram further illustrating the relationships between representative DEPs and selected GO terms is provided in Figure S2.

### 3.4. Subcellular Localization Prediction of Identified Proteins and Protein–Protein Interaction (PPI) Analysis of DEPs

To further characterize the proteomic dataset and explore potential functional associations among DEPs, we performed PSORTb-based subcellular localization prediction and STRING-based PPI network analysis.

#### 3.4.1. Subcellular Localization Distribution

Subcellular localization prediction of all identified proteins showed that most proteins were assigned to the cytoplasm, whereas only small subsets were predicted to be associated with the plasma membrane or extracellular compartment (Figure S4; Table S5). Specifically, 3306 proteins were assigned to the cytoplasm, 18 to the plasma membrane, and 11 to the extracellular compartment. This distribution indicates that the DIA-based proteomic dataset was dominated by cytoplasmic proteins, suggesting that the detected proteomic response mainly reflected intracellular metabolic and stress-response processes. However, because this analysis was performed for all identified proteins, the localization pattern should not be overinterpreted as being specific to DEPs. In addition, the predominance of cytoplasmic proteins may partly reflect a technical bias of the extraction and LC-MS workflow toward soluble proteins. Because no membrane-enrichment strategy was used, membrane-associated and highly hydrophobic proteins may be underrepresented in the present dataset. Therefore, the subcellular localization results should be interpreted cautiously, especially for membrane transport, cell-envelope remodeling, and other membrane-associated responses.

#### 3.4.2. Topological Analysis of the PPI Network

**Figure 3:**
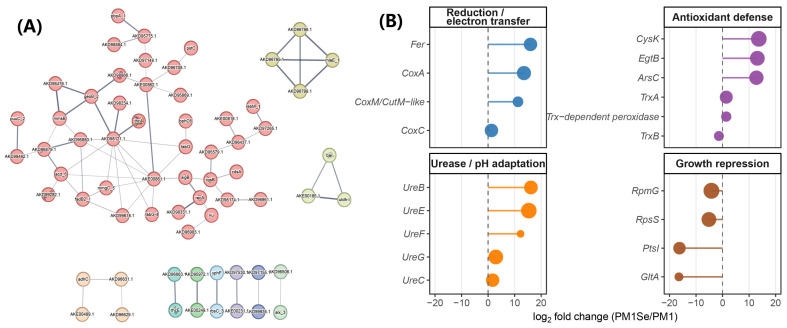
Network organization and representative response modules of differentially expressed proteins (DEPs) in *R. qingshengii* PM1 under selenite stress. (**A**) STRING-based protein–protein interaction (PPI) network of selected DEPs. Nodes represent proteins, edges represent predicted functional associations, and node colors indicate MCL clusters. (**B**) Faceted lollipop plot showing representative DEPs grouped into four response modules: reduction/electron transfer, antioxidant defense, urease-associated pH adaptation, and growth repression. The x-axis represents log2 fold change (PM1Se/PM1). Positive values indicate proteins with higher abundance in PM1Se, whereas negative values indicate proteins with lower abundance in PM1Se relative to the control.

To examine potential functional associations among DEPs, a STRING-based PPI network was constructed using a medium confidence score threshold of 0.4 (Figure 3A; Table S6). MCL clustering divided the selected DEPs into several functional clusters, suggesting that the selenite-responsive proteins were organized into functionally related groups rather than isolated individual proteins. One cluster contained ribosome-associated proteins such as rpsB, rpsJ, and rpsS, consistent with the KEGG enrichment of the ribosome pathway and the coordinated repression of translation-related proteins under selenite stress. Other clusters included proteins associated with DNA repair, lipid metabolism, redox processes, and cell-envelope-related functions, such as recA, fabG, and fadB2. These predicted associations suggest that PM1 may coordinate translation control, repair processes, and metabolic adjustment during adaptation to selenite stress. Overall, the PPI network provides a systems-level view of potential functional coordination among selenite-responsive proteins. Together with the representative protein abundance changes shown in Figure 3B, these results support the presence of multiple response modules, including translation repression, redox adjustment, antioxidant defense, urease-associated adaptation, and structural repair.

### 3.5. Core Selenite-Stress Response Modules of Strain PM1

**Figure 4:**
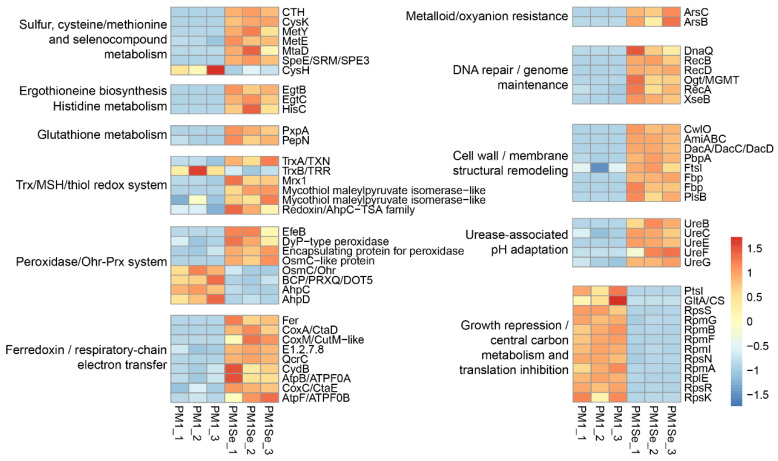
Heatmap of representative selenite-stress-related differentially expressed proteins in *R. qingshengii* PM1. The heatmap shows the relative abundance patterns of selected differentially expressed proteins associated with major selenite-response modules, including sulfur, cysteine/methionine and selenocompound metabolism; ergothioneine biosynthesis; GSH-associated metabolism; Trx/MSH thiol-redox systems; peroxidase/Ohr-Prx detoxification; ferredoxin/respiratory-chain electron transfer; metalloid/oxyanion resistance; DNA repair and genome maintenance; cell wall/membrane structural remodeling; urease-associated pH adaptation; and growth repression involving central carbon metabolism and translation inhibition. Columns represent three biological replicates of the control group (PM1_1–PM1_3) and the selenite-treated group (PM1Se_1–PM1Se_3). The color scale indicates row-normalized protein abundance, with red representing higher relative abundance and blue representing lower relative abundance.

To obtain a more comprehensive view of the adaptive strategy of strain PM1 under high-concentration selenite stress, 68 selenite-stress-related DEPs were selected from the statistically defined DEP dataset for detailed mechanistic interpretation. The selection was based on two objective criteria: first, the proteins met the differential-expression threshold used in this study; second, their functional annotations in GO, KEGG, EggNOG, Pfam, or COG were associated with selenium/metal(loid) stress responses or major stress-adaptation processes, including redox adjustment, sulfur metabolism, antioxidant defense, metalloid/oxyanion resistance, urease-associated pH adaptation, DNA repair, cell-envelope remodeling, and growth repression (Figure 4 and Table S7). These proteins were selected for focused visualization and interpretation, whereas the global DEP identification and enrichment analyses were performed using the complete DEP dataset. These proteins collectively indicate that PM1 does not respond to selenite stress through a single reductase or isolated antioxidant enzyme, but instead activates an integrated metabolic response network.

#### 3.5.1. Ferredoxin-Associated Redox Adjustment and Respiratory-Chain Remodeling

A prominent feature of the PM1 response was the strong induction of proteins associated with electron transfer and respiratory-chain remodeling. Ferredoxin (fer) showed one of the highest abundance increases in the dataset, with a log_2_FC of 15.97, suggesting that ferredoxin may serve as an important electron-transfer component during the selenite stress response. In parallel, several respiratory and redox-associated proteins were upregulated, including cytochrome c oxidase subunit I coxA/ctaD, cytochrome c oxidase subunit III coxC/ctaE, a FAD-binding oxidoreductase-like protein annotated as coxM/cutM, qcrC, cydB, and ATP synthase-associated proteins atpB and atpF. A pyruvate ferredoxin/flavodoxin oxidoreductase-like protein was also induced, further supporting the remodeling of intracellular electron-transfer capacity. The coordinated induction of ferredoxin, terminal oxidase components, cytochrome-associated proteins, and ATP synthase-related proteins suggests that PM1 activates a broad redox-adjustment module under selenite exposure. This module may contribute to electron delivery, respiratory adaptation, and maintenance of redox balance during Se(IV) reduction. However, because the present evidence is based on comparative proteomics, the direct electron-transfer route from ferredoxin or respiratory-chain proteins to selenite reduction requires further biochemical validation.

#### 3.5.2. Sulfur, Cysteine/Methionine, and Selenocompound Metabolism

Selenite stress also triggered extensive remodeling of sulfur-related metabolism. Several proteins involved in cysteine/methionine and selenocompound metabolism were strongly induced, including cysteine synthase A cysK, cystathionine γ-lyase-like CTH, metY, metE, mtaD, and speE, whereas cysH was downregulated (Table S7). Among these proteins, cysK showed a strong increase in abundance, indicating enhanced cysteine-associated sulfur flux. CTH and metE were annotated in selenocompound metabolism, suggesting that sulfur and selenium metabolic routes may be functionally coupled during selenite adaptation. These changes indicate that PM1 may enhance sulfur-associated precursor supply to support thiol metabolism, redox buffering, and maintenance of sulfur-dependent redox proteins. The induction of cysK, CTH, metY, metE, mtaD, and speE suggests that cysteine/methionine metabolism is not a peripheral response, but a central component of PM1 adaptation to selenite stress. This sulfur-associated metabolic remodeling provides a biochemical foundation for the downstream activation of thiol-redox defense systems.

#### 3.5.3. Antioxidant and Thiol-Redox Defense

A major adaptive feature of PM1 was the activation and differential remodeling of multiple antioxidant and thiol-redox systems. First, proteins associated with ergothioneine biosynthesis were strongly induced. In addition to egtB, egtC and hisC were also upregulated, suggesting activation of multiple components of the ergothioneine biosynthetic route rather than a single antioxidant-related protein. This pattern supports the involvement of ergothioneine-associated specialized antioxidant defense under severe selenite stress.

Second, several GSH-associated metabolic proteins were upregulated, including pxpA, speE, and pepN. Although canonical glutathione biosynthetic enzymes such as gshA and gshB were not identified as major DEPs, the induction of these GSH-associated proteins suggests that glutathione-related metabolic processes may contribute to redox buffering or amino acid/thiol metabolism. Therefore, PM1 appears to engage GSH-associated metabolism, but the complete activation of a canonical glutathione cycle cannot be concluded from the present proteomic data alone.

Third, PM1 remodeled thioredoxin- and mycothiol-associated redox systems. TrxA and a redoxin/AhpC-TSA family protein were upregulated, whereas trxB/TRR showed decreased abundance. This opposite regulation suggests that the thioredoxin system was differentially adjusted rather than uniformly activated. In addition, mycothiol-related proteins, including Mrx1 and mycothiol-dependent maleylpyruvate isomerase-like proteins, were induced. Because mycothiol is a characteristic low-molecular-weight thiol in actinobacteria, these results suggest that PM1 may partly rely on an actinomycete-specific MSH-dependent redox buffering system during selenite stress.

Fourth, peroxidase- and organic hydroperoxide resistance-associated proteins were differentially regulated. EfeB, a DyP-type peroxidase-related protein, an OsmC-like protein, and an encapsulating protein for peroxidase were upregulated, whereas several AhpC/Prx-related proteins, including AhpC, AhpD, and BCP/PRXQ-like proteins, were downregulated. This contrasting pattern indicates that PM1 did not simply activate all peroxide-scavenging systems uniformly. Instead, selenite stress induced selective remodeling of peroxidase/Ohr-Prx-related components. Together, these results show that PM1 deploys a layered antioxidant architecture composed of sulfur-associated thiol metabolism, ergothioneine biosynthesis, GSH-associated metabolism, Trx/MSH redox buffering, and peroxidase/Ohr-Prx-related detoxification. This antioxidant network provides a broader and more specific explanation for oxidative-stress adaptation than a simple SOD/POD-centered model.

#### 3.5.4. Metalloid/Oxyanion Resistance and Detoxification-Associated Proteins

In addition to thiol-redox defense, PM1 induced proteins associated with metalloid or oxyanion resistance. The arsenate reductase-like protein arsC was strongly upregulated, and arsB was also induced. Although these proteins are classically associated with arsenic resistance, their induction under selenite exposure suggests activation of a broader metalloid/oxyanion stress-response module. This module may contribute to intracellular detoxification, redox adjustment, or efflux-associated stress adaptation. However, the direct substrate specificity of ArsC/ArsB toward selenium oxyanions remains to be experimentally verified. The simultaneous induction of arsC/arsB and thiol-redox proteins suggests that metalloid resistance and antioxidant defense are functionally connected in PM1. In particular, ArsC-like reductases often depend on thiol-based electron donors, and their induction is consistent with the observed remodeling of Trx/MSH-associated systems.

#### 3.5.5. Urease-Associated pH Adaptation

A pronounced urease-associated module was also activated under selenite stress. UreB was among the most strongly upregulated proteins, and this response was accompanied by increased abundance of ureC, ureE, ureF, and ureG. The coordinated induction of both structural and accessory urease proteins suggests activation of the urease machinery as a functional module rather than the isolated upregulation of a single subunit. This urease-associated response may contribute to ammonia-mediated pH buffering and intracellular homeostasis under selenite stress. Because selenite exposure and reduction may disturb proton balance and impose acid/homeostasis stress, urease-mediated ammonia production could help maintain intracellular conditions favorable for detoxification enzymes and redox metabolism. Nevertheless, direct measurements of urease activity, ammonia production, and cytosolic pH are required to validate this proposed mechanism.

#### 3.5.6. DNA Repair and Genome Maintenance

Selenite stress also induced a set of proteins associated with DNA repair and genome maintenance. Homologous recombination-related proteins, including recA, recB, and recD, were upregulated. In addition, dnaQ, ogt/MGMT, and xseB were induced, indicating activation of multiple DNA repair routes, including replication proofreading, alkylation damage repair, mismatch repair, and recombination-associated repair. The induction of these proteins is consistent with the oxidative and genotoxic pressure generated during selenite stress. Reactive oxygen species produced during Se(IV) reduction can damage DNA and other macromolecules, and the upregulation of RecA/RecBCD-associated repair components suggests that strain PM1 activates genome maintenance mechanisms to preserve cellular viability under high selenium load.

#### 3.5.7. Cell Wall and Membrane Structural Remodeling

Proteins involved in cell wall and membrane remodeling were also strongly induced. The cell-wall hydrolase cwlO and N-acetylmuramoyl-L-alanine amidase amiABC showed high abundance increases, suggesting active remodeling of peptidoglycan or cell-envelope architecture. Additional envelope-associated proteins, including dacA/dacC/dacD, pbpA, ftsI, fbp, and plsB, were also upregulated. These proteins are associated with peptidoglycan remodeling, cell-wall maintenance, arabinogalactan-related processes, glycerolipid/glycerophospholipid metabolism, and membrane structural adaptation. Their induction suggests that strain PM1 reinforces envelope repair and membrane remodeling under selenite stress, likely to counteract oxidative damage, maintain cell integrity, and regulate permeability to toxic oxyanions.

#### 3.5.8. Growth Repression, Central Carbon Metabolic Adjustment, and Translation Inhibition

In contrast to the strong induction of defense- and repair-associated proteins, strain PM1 showed marked repression of proteins associated with growth, carbon uptake, central metabolism, and translation. The phosphotransferase system component ptsI was strongly downregulated, suggesting reduced carbohydrate uptake or sugar-phosphotransfer activity. Citrate synthase gltA, a key enzyme of the tricarboxylic acid cycle, was also strongly suppressed, indicating reduced central aerobic carbon metabolism. Translation-related proteins were broadly downregulated. Representative ribosomal proteins, including rpsS, rpmG, rpmB, rpmF, rpmI, rpsN, rpmA, rplF, rpsR, and rpsK, showed significant decreases in abundance. This coordinated repression is consistent with the significant enrichment of the ribosome pathway in KEGG analysis and supports the interpretation that strain PM1 enters a growth-restrictive, energy-conserving state under severe selenite stress.

## 4. Discussion

### 4.1. Systems-Level Proteomic Reprogramming in Strain PM1

At such high selenite stress, strain PM1 survival is unlikely to rely solely on a few resistance genes. Instead, it appears to rely on whole-cell proteomic reprogramming. The large number of DEPs and the clear separation between PM1 and PM1Se indicate that selenite exposure reshaped the cellular allocation of proteins from growth-associated functions toward detoxification, redox homeostasis, structural repair, and intracellular adaptation (Figure 4). Similar systems-level responses have been reported in Stenotrophomonas bentonitica BII-R7, where cells mobilize metabolic and repair functions to counteract metal-induced oxidative stress [8]. Broad changes in protein synthesis have also been observed in Rhodobacter sphaeroides under selenium stress [26]. In addition, proteomics studies have suggested that selenite stress may trigger threshold-dependent network rewiring rather than a gradual single-pathway response [27]. Together, these observations support the view that strain PM1 tolerance arises from integrated regulatory and metabolic adaptation rather than from the isolated action of a single reductase. In this context, 68 selenite-stress-related DEPs should be viewed not as isolated markers, but as evidence for a coordinated stress-adaptation architecture involving redox balancing, thiol metabolism, intracellular homeostasis, structural repair, and growth repression.

### 4.2. Ferredoxin-Associated Redox Adjustment May Contribute to Selenium Detoxification

A central feature of strain PM1 response was the activation of a ferredoxin-associated redox module. Unlike selenium-reducing systems in which cytochrome- or flavin-dependent reductases are often emphasized [28], such as those reported in Shewanella oneidensis MR-1 [29], strain PM1 showed a particularly strong induction of ferredoxin together with respiratory-chain and oxidoreductase-associated proteins. Ferredoxin has a very low redox potential and is therefore thermodynamically suitable for participating in reduction reactions involving high-valence metal(loid)s [30]. This suggests that ferredoxin may contribute to the electron-transfer environment required for Se(IV) reduction or redox balancing under selenite stress. However, the PM1 response should not be interpreted as a simple ferredoxin-only mechanism. The simultaneous induction of respiratory-chain components, terminal oxidase-associated proteins, FAD-binding oxidoreductase-like proteins, and ATP synthase-related proteins suggests broader respiratory-chain remodeling. Such remodeling may help maintain electron flow, respiratory flexibility, and energy availability under oxidative stress. Thus, the ferredoxin-associated module is better interpreted as part of a redox-adjustment network that may support selenium detoxification while also maintaining intracellular redox balance [31]. This interpretation remains a proteomics-based hypothesis. The current data shows strong protein-level associations, but they do not directly prove electron transfer from ferredoxin to selenite. Biochemical assays, redox-interaction experiments, and genetic validation will be required to determine whether ferredoxin directly participates in Se(IV) reduction or functions indirectly within a broader stress-associated electron-transfer network.

### 4.3. Sulfur Metabolism and Thiol-Redox Defense Under Selenite Stress

Selenite reduction can generate ROS, and ROS can damage thiol groups and iron–sulfur proteins [32,33]. Fisher et al. showed that superoxide generated during selenite reduction can attack Fe–S clusters in ferredoxin, leading to enzyme inactivation [33]. This provides a mechanistic context for the strong remodeling of sulfur-related metabolism observed in strain PM1. Because selenium and sulfur share chemical similarities, microbial selenite responses are often closely linked to sulfur assimilation, thiol metabolism, and oxidative-stress responses [10].

In strain PM1, the induction of proteins related to cysteine/methionine and selenocompound metabolism suggests reinforcement of thiol precursor supply. This sulfur-centered response may help provide cysteine-derived reducing capacity for thiol buffering, repair of oxidized proteins, and maintenance of Fe–S-dependent redox proteins [34,35,36]. Although the present data do not directly demonstrate activation of the Isc/Suf Fe–S assembly machinery, enhanced sulfur precursor supply is consistent with the known requirement for cysteine-derived sulfur in Fe–S cluster biogenesis and repair [35,36].

Beyond precursor supply, PM1 appears to deploy a layered antioxidant defense rather than relying on a single classical antioxidant enzyme system. The induction of ergothioneine biosynthesis-related proteins is particularly noteworthy. Ergothioneine is a histidine-derived sulfur-containing antioxidant whose biosynthesis is mediated by egt genes, with EgtB/EgtD regarded as key enzymes in the bacterial pathway [37]. Because the canonical egt gene cluster is particularly associated with Actinobacteria, the induction of egtB and egtC in PM1 is consistent with activation of an actinobacteria-associated ergothioneine biosynthetic route [38]. Compared with glutathione, ergothioneine is more stable under physiological conditions and less prone to auto-oxidation [39]. It can also protect proteins from oxidative and nitrosative damage, including protein tyrosine nitration induced by reactive nitrogen species [40]. Although ergothioneine itself was not quantified in this study, the proteomic evidence suggests that PM1 may activate this specialized antioxidant route under severe selenium stress.

The antioxidant response also involved GSH-associated metabolism, Trx/MSH thiol-redox systems, and peroxidase/Ohr-Prx-related proteins. This is especially relevant for Rhodococcus, because actinobacteria often rely on mycothiol rather than glutathione as a major low-molecular-weight thiol [41]. The remodeling of Trx- and MSH-associated components suggests that strain PM1 uses actinomycete-specific thiol buffering to cope with oxidative stress. The Trx system is a widely conserved redox regulatory system involved in maintaining protein thiol status and oxidative-stress resistance [42]. In strain PM1, the contrasting regulation of different Trx- and peroxidase-associated components indicates selective remodeling rather than uniform activation of all antioxidant enzymes. Therefore, the antioxidant strategy of PM1 is best described as a multi-layered thiol-redox defense network. This network integrates sulfur-associated precursor supply, ergothioneine biosynthesis, GSH-associated metabolism, Trx/MSH redox buffering, and peroxidase/Ohr-Prx-related detoxification. Such a flexible antioxidant architecture may be particularly advantageous under high selenite pressure, where ROS generation, thiol oxidation, and redox imbalance occur simultaneously.

### 4.4. Metalloid/Oxyanion Resistance, pH Adaptation, and Intracellular Homeostasis

In addition to antioxidant defense, PM1 activated proteins associated with metalloid/oxyanion resistance. The induction of ArsC/ArsB-related components suggests that PM1 may recruit broad oxyanion-response systems under selenite stress. Although ArsC and ArsB are classically associated with arsenic resistance, arsenic and selenium oxyanions share overlapping features in transport, redox chemistry, and microbial detoxification responses [43,44]. Moreover, selenite and related chalcogen oxyanions may interact with cellular thiol chemistry and electron-transfer systems after entering the cell, linking oxyanion resistance to redox homeostasis [43]. The co-occurrence of ArsC/ArsB induction with Trx/MSH remodeling further suggests that metalloid resistance and thiol-redox buffering may be functionally connected, because ArsC-type arsenate reductases commonly rely on thiol-based redox cascades involving glutaredoxin, glutathione, or thioredoxin systems [45,46]. Nevertheless, direct selenium-related roles of ArsC or ArsB remain to be experimentally confirmed.

Another prominent adaptive feature was urease-associated pH adaptation. Urease hydrolyzes urea to generate ammonia and carbon dioxide, and ammonia production can increase intracellular and extracellular pH, thereby contributing to acid resistance and pH homeostasis in microorganisms [47,48]. Under selenite stress, oxyanion uptake, redox reactions, and metabolic disturbance may perturb proton balance and impose acid/homeostasis stress, consistent with the reported links between chalcogen oxyanion transport, membrane energetics, and redox imbalance in bacteria [43]. Urease-derived NH_3_ may therefore help maintain intracellular conditions favorable for detoxification enzymes and redox metabolism. The coordinated induction of urease structural and accessory proteins supports the idea that PM1 activates urease as a functional homeostasis module rather than as an isolated nitrogen-metabolism enzyme. This urease-associated response may be particularly important under high-concentration selenite stress, where redox reactions, ROS detoxification, and repair processes impose high physiological costs. However, the proposed pH-buffering function remains inferential. Direct measurements of urease activity, ammonia production, extracellular pH, and cytosolic pH are required to validate this mechanism.

### 4.5. DNA Repair and Cell-Envelope Remodeling as Structural Protection Layers

Selenite exposure can induce oxidative/redox stress, and excessive ROS can damage DNA, proteins, membrane lipids, and envelope-associated components [32]. The induction of DNA repair-associated proteins in PM1 suggests that genome maintenance is an important component of its selenite response. RecA and RecBCD-related systems are central to homologous recombination and repair of oxidative DNA damage in bacteria, while proteins involved in proofreading, alkylation repair, and exonuclease processing may further contribute to genome stability [49,50]. Such responses are consistent with the need to repair oxidative or genotoxic damage generated under severe selenite stress.

In parallel, strain PM1 also appears to reinforce its cell envelope. Proteins associated with peptidoglycan remodeling, penicillin-binding functions, arabinogalactan-related processes, and glycerolipid/glycerophospholipid metabolism were induced, suggesting active remodeling of the cell wall and membrane. Cell-envelope remodeling can help preserve permeability barriers, maintain structural stability, and reduce damage caused by oxidative or metal(loid) stress [51,52]. Peptidoglycan remodeling has also been shown to enhance bacterial envelope robustness under envelope stress conditions [53]. These structural protection modules likely function together with antioxidant systems. Antioxidant proteins reduce oxidative pressure, DNA repair proteins maintain genome integrity, and envelope remodeling proteins preserve the physical barrier required for sustained detoxification. This combination may be essential for maintaining viability during prolonged exposure to high selenite concentrations. It should also be noted that the current extraction workflow was not specifically optimized for membrane-protein enrichment. Therefore, membrane-associated proteins involved in transport, envelope remodeling, or oxyanion uptake may be underrepresented. This limitation may partly affect the interpretation of membrane- and cell-envelope-related responses, and future studies using membrane-enriched proteomics or targeted validation will be required to further resolve these processes.

### 4.6. Growth–Defense Trade-Off and Resource Reallocation

The activation of detoxification, antioxidant defense, pH buffering, DNA repair, and envelope remodeling requires substantial energy and biosynthetic investment. Strain PM1 appears to balance this cost by repressing growth-associated functions. The downregulation of central carbon metabolism and carbohydrate uptake-related proteins suggests reduced biomass-generating metabolism, while the repression of ribosomal proteins indicates reduced translational capacity. Protein biosynthesis is one of the most energy-intensive cellular processes, and ribosome synthesis and translational capacity are tightly coupled to bacterial growth status [54,55]. Reducing ribosome biogenesis and translation can conserve ATP, amino acids, and carbon resources under stress. It may also reduce the biosynthetic and proteostasis burden associated with protein production, folding, transport, and repair during oxidative stress [55,56]. In addition, downregulation of protein biosynthesis has been proposed to liberate energy and cellular resources under environmental stress conditions [57]. Ribosome-profiling studies further show that bacteria can regulate translational efficiency in response to environmental stresses, including osmotic and acid stress, thereby helping balance survival and growth [58,59]. Therefore, the PM1 response can be interpreted as a growth–defense trade-off. Under selenite stress, PM1 reduces investment in growth, translation, carbohydrate uptake, and central carbon metabolism, while redirecting cellular resources toward redox adjustment, sulfur-associated thiol metabolism, layered antioxidant defense, metalloid/oxyanion resistance, urease-associated pH buffering, DNA repair, and envelope remodeling. This coordinated reallocation likely enables PM1 to maintain detoxification capacity and structural integrity while limiting the energetic burden of growth.

**Figure 5:**
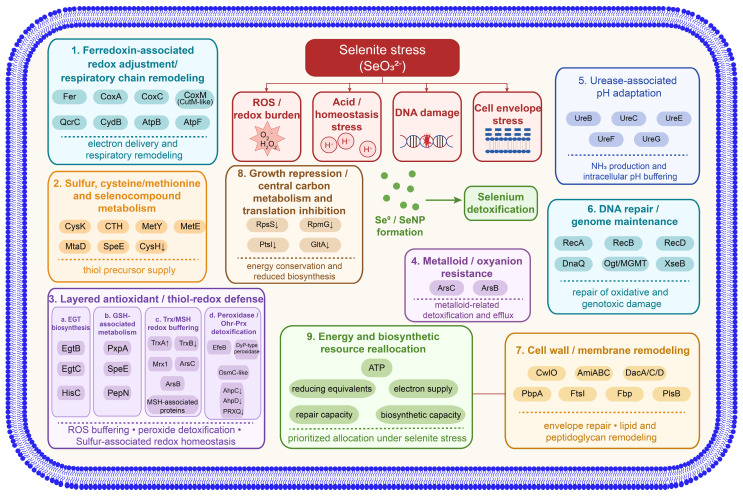
Proposed metabolic response model of *R. qingshengii* PM1 under selenite stress. DIA-based proteomic data suggest that PM1 survives selenite stress through a growth–defense trade-off strategy, involving ferredoxin-associated redox adjustment, sulfur-associated thiol metabolism, antioxidant defense, urease-associated pH buffering, DNA repair, cell envelope remodeling, and repression of growth-associated metabolism.

Based on these findings, we propose an integrated metabolic response model for PM1 under selenite stress (Figure 5). In this model, SeO_3_^2−^ exposure imposes ROS/redox burden, acid/homeostasis stress, DNA damage, and cell-envelope stress. PM1 responds by activating ferredoxin-associated electron transfer, sulfur-linked thiol precursor supply, antioxidant defense, metalloid/oxyanion resistance, urease-associated pH buffering, genome maintenance, and cell wall/membrane remodeling. At the same time, repression of translation and central carbon metabolism conserves energy and biosynthetic resources. This systems-level strategy allows PM1 to prioritize survival and detoxification over rapid proliferation.

## 5. Conclusions

In summary, this study provides a systems-level view of the proteomic response of *R. qingshengii* PM1 to high-concentration selenite stress. DIA-based quantitative proteomics revealed that PM1 reallocates protein resources from growth-associated processes toward detoxification, redox homeostasis, intracellular adaptation, and structural repair. The curated 68 selenite-stress-related DEPs suggest a coordinated multi-module response involving ferredoxin-associated redox adjustment, sulfur-linked thiol metabolism, antioxidant defense, metalloid/oxyanion resistance, urease-associated pH adaptation, DNA repair, cell-envelope remodeling, and growth repression. Rather than supporting a simple single-reductase model, the data suggest that PM1 relies on a broader redox-adjustment network. These findings expand the understanding of selenium-related stress responses in Rhodococcus and provide candidate targets for future functional validation and strain engineering for selenium/metal(loid) bioremediation.

## Data Availability

The raw DIA mass spectrometry data generated in this study have been deposited in iProX. The dataset is available under the iProX project accession IPX0017907000 and subproject accession IPX0017907001. The mass spectrometry proteomics data have been deposited to the ProteomeXchange Consortium (https://proteomecentral.proteomexchange.org, accessed on 23 June 2026) via the iProX partner repository with the dataset identifier PXD080037.

## Supplementary Materials

The following supporting information can be downloaded at: https://www.mdpi.com/article/10.3390/microorganisms14071455/s1, Figure S1. Quality assessment of DIA-based proteomic identification data. (A) Peptide length distribution of all identified peptides. The x-axis represents peptide length in amino acid residues, and the y-axis represents the number of identified peptides. Most peptides were distributed between 7 and 20 amino acids, which is consistent with the expected cleavage characteristics of trypsin digestion. (B) Distribution of peptide numbers per identified protein. The x-axis represents the number of peptides assigned to each protein, and the y-axis represents the number of proteins. The identification of proteins supported by multiple peptides indicates the reliability of the proteomic dataset. Figure S2. GO chord diagram illustrating the relationships between representative differentially expressed proteins (DEPs) and enriched GO terms in *Rhodococcus qingshengii* PM1 under selenite stress. Ribbons indicate protein–term associations, and the color scale represents log2 fold change (PM1Se/PM1). Major enriched terms include translation, amide biosynthetic process, peptide biosynthetic process, peptide metabolic process, amide metabolic process, macromolecule biosynthetic process, protein metabolic process, amide transport, peptide transport, and xenobiotic metabolic process. Figure S3. Hierarchical clustering heatmap of selected differentially expressed proteins in *Rhodococcus qingshengii* PM1 under selenite stress. Columns represent biological replicates from the control group (PM1) and the selenite-treated group (PM1Se), and rows represent individual proteins. Color intensity indicates relative protein abundance after row-wise normalization, showing clear separation between the two groups. Figure S4. Predicted subcellular localization distribution of all identified proteins in *Rhodococcus qingshengii* PM1 based on PSORTb annotation. Most identified proteins were predicted to localize in the cytoplasm (CYT), whereas smaller subsets were assigned to the plasma membrane (PLA) or extracellular compartment (EXC). Table S1: Protein identification and quantification matrix obtained from DIA-based proteomic analysis of *Rhodococcus qingshengii* PM1 under selenite stress; Table S2: Differentially expressed proteins identified in *Rhodococcus qingshengii* PM1 under selenite stress; Table S3: KEGG pathway enrichment analysis of differentially expressed proteins in *Rhodococcus qingshengii* PM1 under selenite stress; Table S4: GO functional classification of differentially expressed proteins in *Rhodococcus qingshengii* PM1 under selenite stress; Table S5: PSORTb-based subcellular localization prediction of all identified proteins in *Rhodococcus qingshengii* PM1; Table S6: MCL clustering and module composition of the STRING-based PPI network of selected DEPs under selenite stress; Table S7: *Rhodococcus qingshengii* PM1 selenite stress related protein summary.
