# Supplementary material for: DIA-Based Quantitative Proteomics Reveals Adaptive Responses and Potential Mechanisms of Se(IV) Resistance in Rhodococcus qingshengii PM1
Source: Microorganisms. 2026 Jul 1;14(7):1455. doi: 10.3390/microorganisms14071455 (PMC13414329; doi:10.3390/microorganisms14071455)
Supplement: Supplementary file 1 [file microorganisms-14-01455-s001.zip › Figue S1.pdf]

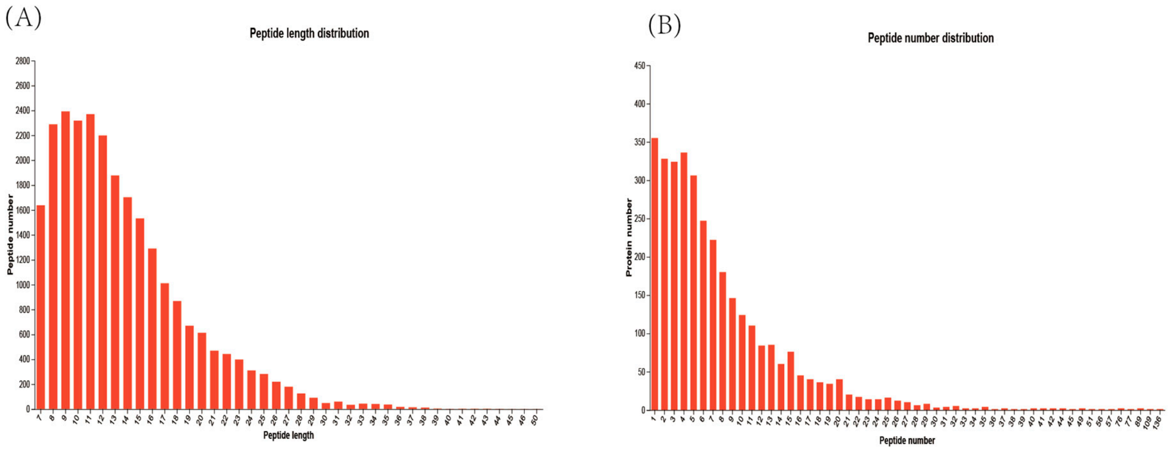

Figure S1. Quality assessment of DIA-based proteomic identification data.

(A) Peptide length distribution of all identified peptides. The x-axis represents peptide length in amino acid residues, and the y-axis represents the number of identified peptides. Most peptides were distributed between 7 and 20 amino acids, which is consistent with the expected cleavage characteristics of trypsin digestion.

(B) Distribution of peptide numbers per identified protein. The x-axis represents the number of peptides assigned to each protein, and the y-axis represents the number of proteins. The identification of proteins supported by multiple peptides indicates the reliability of the proteomic dataset.
