# Supplementary material for: DIA-Based Quantitative Proteomics Reveals Adaptive Responses and Potential Mechanisms of Se(IV) Resistance in Rhodococcus qingshengii PM1
Source: Microorganisms. 2026 Jul 1;14(7):1455. doi: 10.3390/microorganisms14071455 (PMC13414329; doi:10.3390/microorganisms14071455)
Supplement: Supplementary file 1 [file microorganisms-14-01455-s001.zip › Figue S2.pdf]

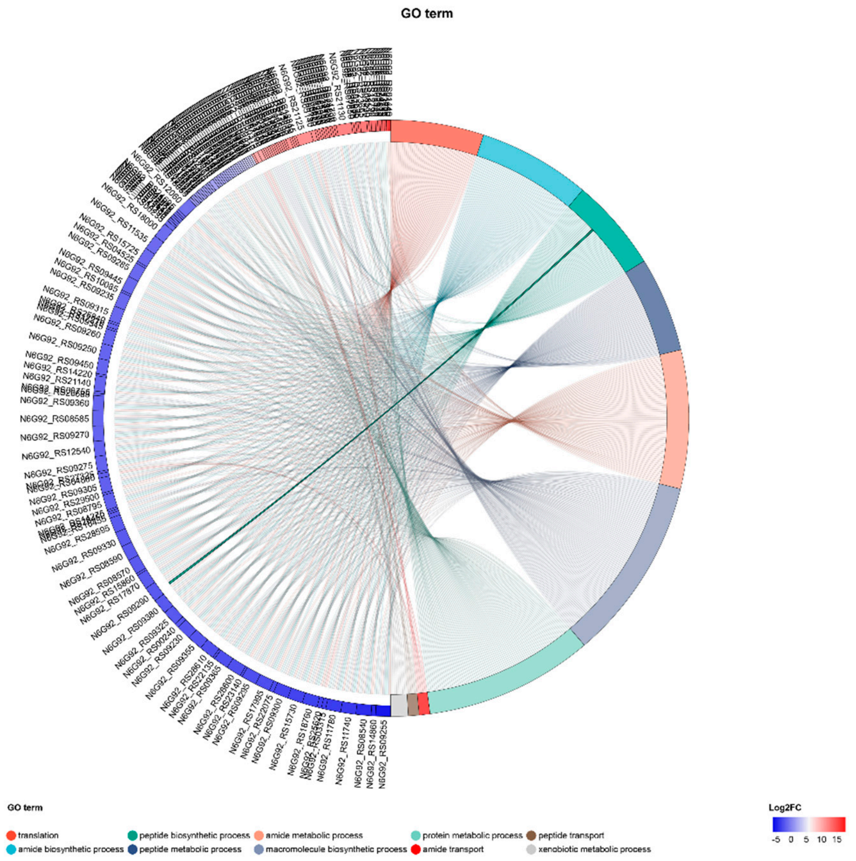

Figure S2. GO chord diagram illustrating the relationships between representative differentially expressed proteins (DEPs) and enriched GO terms in *Rhodococcus qingshengii* PM1 under selenite stress. Ribbons indicate protein–term associations, and the color scale represents log2 fold change (PM1Se/PM1). Major enriched terms include translation, amide biosynthetic process, peptide biosynthetic process, peptide metabolic process, amide metabolic process, macromolecule biosynthetic process, protein metabolic process, amide transport, peptide transport, and xenobiotic metabolic process.
