# Supplementary material for: DIA-Based Quantitative Proteomics Reveals Adaptive Responses and Potential Mechanisms of Se(IV) Resistance in Rhodococcus qingshengii PM1
Source: Microorganisms. 2026 Jul 1;14(7):1455. doi: 10.3390/microorganisms14071455 (PMC13414329; doi:10.3390/microorganisms14071455)
Supplement: Supplementary file 1 [file microorganisms-14-01455-s001.zip › Figue S3.pdf]

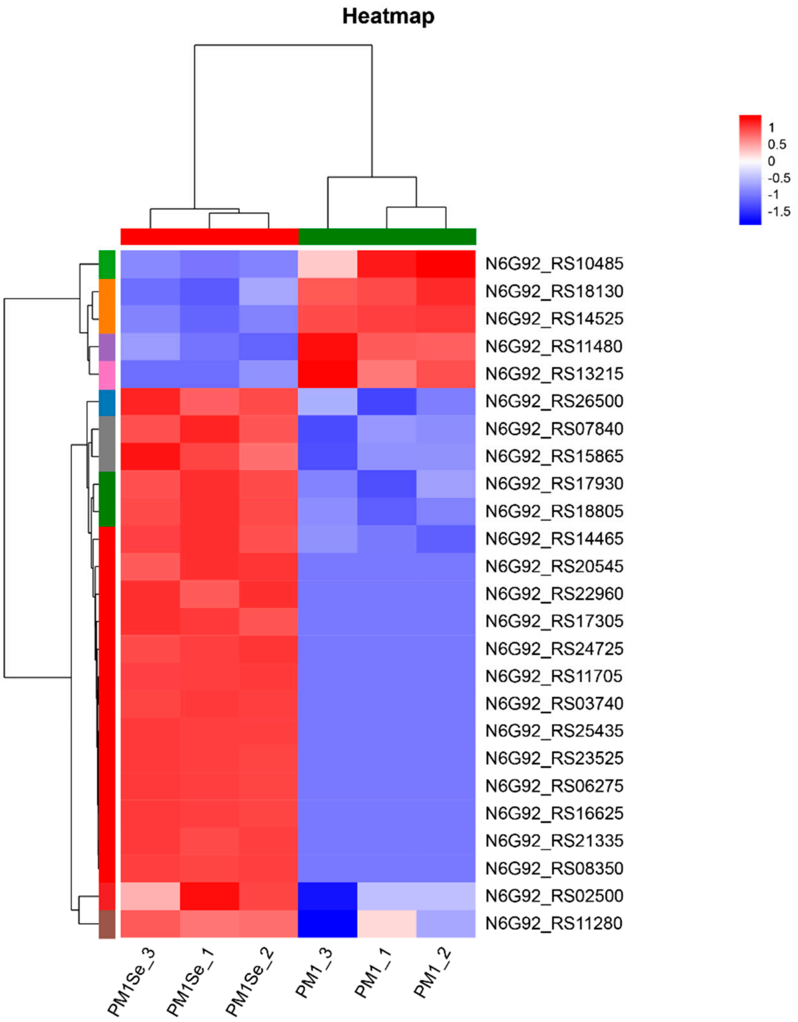

Figure S3. Hierarchical clustering heatmap of selected differentially expressed proteins in *Rhodococcus qingshengii* PM1 under selenite stress. Columns represent biological replicates from the control group (PM1) and the selenite-treated group (PM1Se), and rows represent individual proteins. Color intensity indicates relative protein abundance after row-wise normalization, showing clear separation between the two groups.
