# Supplementary material for: DIA-Based Quantitative Proteomics Reveals Adaptive Responses and Potential Mechanisms of Se(IV) Resistance in Rhodococcus qingshengii PM1
Source: Microorganisms. 2026 Jul 1;14(7):1455. doi: 10.3390/microorganisms14071455 (PMC13414329; doi:10.3390/microorganisms14071455)
Supplement: Supplementary file 1 [file microorganisms-14-01455-s001.zip › Figue S4.pdf]

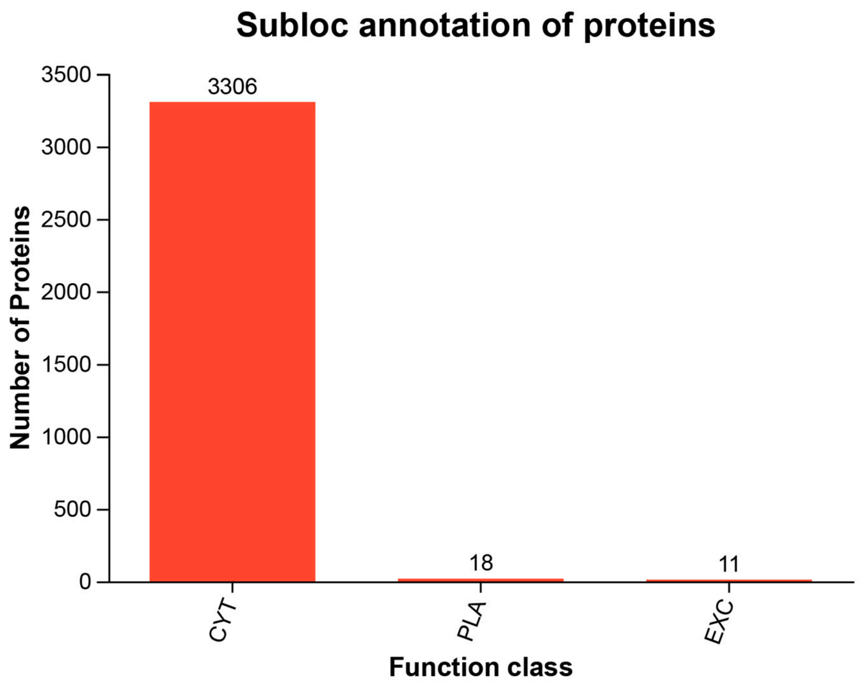

Figure S4. Predicted subcellular localization distribution of all identified proteins in *Rhodococcus qingshengii* PM1 based on PSORTb annotation. Most identified proteins were predicted to localize in the cytoplasm (CYT), whereas smaller subsets were assigned to the plasma membrane (PLA) or extracellular compartment (EXC).
